# Males with Kawasaki disease develop coronary artery aneurysms more than twice as much as females

**DOI:** 10.1093/pch/pxae106

**Published:** 2025-04-03

**Authors:** Laurence Watelle, Andrea Dahoud, Samuel Blais, Rosie Scuccimarri, Claudia Renaud, Brian W McCrindle, Dereck Human, Frédéric Dallaire, Nagib Dahdah

**Affiliations:** Department of Pediatrics, Faculty of Medicine and Health Sciences, Université de Sherbrooke, and Centre de recherche du Centre Hospitalier Universitaire de Sherbrooke, Sherbrooke, Quebec, Canada; Pediatric Cardiology Division, CHU Sainte-Justine, Université de Montréal, Montreal, Quebec, Canada; Department of Pediatrics, Faculty of Medicine and Health Sciences, Université de Sherbrooke, and Centre de recherche du Centre Hospitalier Universitaire de Sherbrooke, Sherbrooke, Quebec, Canada; Department of Pediatrics, Montreal Children’s Hospital, McGill University, Montreal, Quebec, Canada; Department of Pediatrics, Montreal Children’s Hospital, McGill University, Montreal, Quebec, Canada; Labatt Family Heart Centre, The Hospital for Sick Children, Department of Pediatrics, University of Toronto, Toronto, Ontario, Canada; Division of Pediatric Cardiology, British Columbia Children’s Hospital University of British Columbia, Vancouver, British Columbia, Canada; Department of Pediatrics, Faculty of Medicine and Health Sciences, Université de Sherbrooke, and Centre de recherche du Centre Hospitalier Universitaire de Sherbrooke, Sherbrooke, Quebec, Canada; Pediatric Cardiology Division, CHU Sainte-Justine, Université de Montréal, Montreal, Quebec, Canada

**Keywords:** *Biological sex*, *Coronary artery aneuryms*, *Kawasaki disease*

## Abstract

**Objectives:**

Kawasaki disease (KD) is the leading cause of acquired childhood coronary aneurysms (CAA). Males are more affected than females, with lower survival from cardiac events and normalization rates. This study aimed to determine the association between biological sex and CAA risk and evaluate the association with baseline biochemical inflammatory markers by biological sex.

**Methods:**

This multicenter retrospective cohort study involved children ≤10 years old diagnosed with KD in five Canadian centres. Adjusted CAA risk differences between sexes were computed using binomial regression. Associations between inflammatory markers and CAA risk were analyzed using logistic regression with interaction terms between sex and inflammatory markers.

**Results:**

From 2004 to 2015, 1382 patients were diagnosed with KD and 812 (59%) were males. Median age, fever total duration, and fever duration at therapy initiation were similar between the sexes. The cumulative incidence of medium to large (Z ≥ 5) CAA was higher in males [70/812 (8.6%)] compared to females [19/570 (3.3%)], with an adjusted risk difference of 4.6 % (95% confidence interval [CI] 2.1 to 7.1). Large (Z > 10) aneurysms were more prevalent in males (adjusted risk difference of 3.3%, 95% CI 1.7 to 5.0). Most inflammatory markers were positively associated with CAA risk, but the association was not statistically different between sexes.

**Conclusion:**

Males with KD are at higher risk of developing CAA compared to females. The majority of patients were presumed to be prepubertal, suggesting that hormonal influences are unlikely to be a significant factor. Future KD research based on biological sex categorization should focus on patient risk stratification and long-term prognostic evaluation.

## Graphical Abstract

**Graphical Abstract:**
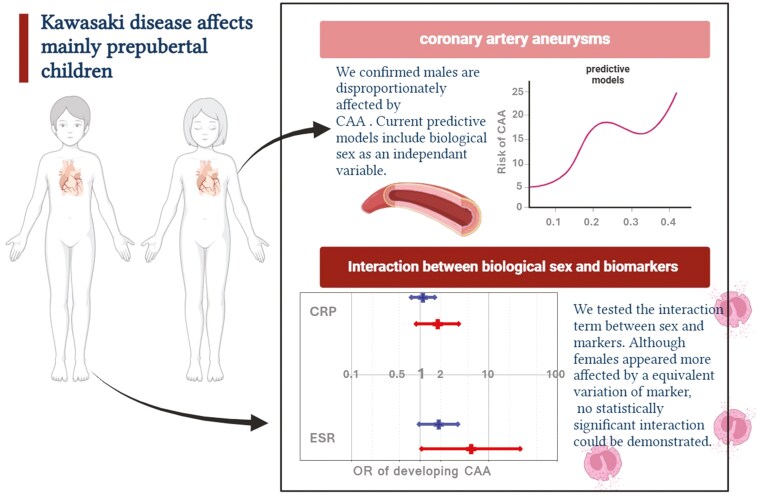

Kawasaki disease (KD) is the commonest acquired pediatric heart disease in high-income countries (1). A feared cardiac complication is the development of coronary artery aneurysms (CAA), which affect approximately 25% of untreated children (1). The etiology of KD remains unclear, but genetic predisposition plays a role; the rate of KD in siblings is 2.1%, with a relative risk of ≈10-fold compared to the general population (2).

Biological sex influences KD pathogenesis, with males affected more frequently (ratio 1.5:1). Studies indicate that males are more prone to develop KD (1) and its coronary artery complications (3,4). Long-term outcomes are worse for males, who face a higher risk of death from cardiac events compared to females (1). The standardized mortality rate of males with coronary artery sequelae is threefold that of females according to the Japanese nationwide series (5). Consequently, sex was included early in clinical prediction tools like the Harada score, developed in 1991 to identify children at risk for CAA due to intravenous immunoglobulin (IVIG) resistance (6). Biochemical predictors such as C-reactive protein (CRP), erythrocyte sedimentation rate (ESR), platelet count, hemoglobin levels, serum albumin, and sodium levels influence CAA risk and are often included in clinical prediction scores like Harada’s (7–9). However, these clinical scores assess each variable independently, and no studies have investigated how different biochemical markers might interact with sex to modify this risk. Additionally, the specific role of biological male sex in relation to aneurysm severity remains inadequately characterized. We aimed to determine the association between biological sex and the risk of developing CAA within 1 year after KD onset. Additionally, we evaluated how biochemical markers at diagnosis differentially affected CAA risk in males compared to females.

## METHODS

### Study design and patient population

This analysis was conducted using secondary data from a retrospective cohort study including all children diagnosed with KD between 2004 and 2015 in five Canadian pediatric tertiary care centres (10). Patient data were retrieved through chart review and echocardiography databases. We included patients ≤10 years of age at the time of diagnosis with complete or incomplete KD, in accordance with the American Heart Association guidelines (1). Eligible subjects were children with a first episode of KD diagnosed and treated with IVIG 2 g/kg. Subjects were eligible if the clinical suspicion of KD was such that treatment with IVIG was deemed clinically indicated by the treating physician. Exclusion criteria were as follows: subjects whose final diagnosis of KD was clearly rejected by the treating physician; subjects with structural cardiac disease at baseline other than cardiac disease secondary to KD; subjects with recurrence of KD (second episode not included); and subjects with follow-up <6 weeks or with incomplete echocardiographic studies during follow-up.

### Independent variable and outcomes

Biological sex as recorded in the medical chart was our primary independent variable. The primary outcome was the cumulative incidence of CAA, defined as any proximal main coronary artery segment, the left main coronary artery (LMCA), left anterior descending artery (LAD), circumflex coronary artery (Cx), or right coronary artery (RCA) with an internal diameter Z score ≥2.5 on at least one echocardiogram within 12 months of KD onset. Small, isolated aneurysms (Z < 5 and ≥2.5) of the left main CA in the acute phase (first 10 days of illness) were excluded as this observation tends to be less clinically significant (1). Secondary outcomes included the cumulative incidence of any CAA, medium or greater CAA (any documentation of internal diameter Z ≥ 5), and large/giant CAA (any documentation of internal diameter Z ≥ 10) within 12 months of KD onset. Coronary artery diameter Z scores were calculated using the formula previously proposed by Dallaire and Dahdah (11).

The secondary objective was to test whether biological sex had a modifying effect on the association of biological markers with the cumulative incidence of CAA. We sought to verify whether the same absolute increase in a marker would further increase the risk of aneurysm in males compared to females. CRP, ESR, platelet count, hemoglobin levels, serum albumin, and natremia were reported at the time of diagnosis in the original dataset. For CRP and ESR, a log transformation was used since these variables had a log-normal distribution. Marker values following a normal distribution were converted to Z scores using age and sex-specific reference values. We compared the impact of biological sex on the risk of CAA for a 2 standard deviation increase in marker values or an exponential factor for log-transformed variables. We determined normal intervals from the CALIPER database, developed from a study of thousands of healthy children and adolescents (12).

### Covariables

Variables tested for possible confounding effects included age at diagnosis (years), fever duration (days), dose of ASA during the acute phase (low versus high), KD type (complete versus incomplete), repeated IVIG treatment, and late IVIG treatment (> 10 days of fever). Ethnicity was not included due to missing data in 21% of the cohort. Low-dose ASA was defined as ≤10 mg/kg/day and high dose as ≥10 mg/kg/day. KD type was defined as per the 2007 AHA guidelines (13).

### Statistical analysis

Descriptive data are presented as medians with interquartile ranges for continuous variables and percentages for categorical variables. Risk differences for CAA between sexes were calculated using a binomial regression model. Variables were included in the final model if the variation in the estimated risk difference (effect size) varied by more than 10% (14). The final variables included in the model were age at diagnosis, repeated treatments, dose of ASA received, and the total duration of fever. A logistic regression model assessed the impact of marker increments on CAA risk, including potential modifying effects of biological sex on the six markers tested. A similar process was applied for the choice of covariables and the same covariables were included. In total, 30.4% of data were missing for CRP, 28.0% for ESR, 23.1% for platelets, 27.2% for hemoglobin, 34.7% for sodium, and 40.1% for albumin. Since the data appeared to be missing completely at random, and given the risk of overfitting, we did not proceed to imputation. For models that did not converge, we sequentially excluded covariables, starting with those least affecting model variation. All statistical analyses were performed in SAS 9.4.

### Ethical considerations

Ethical approval was obtained by the Research Ethics Board of each participating centre. This study made secondary use of anonymous retrospective data. The requirement for patient consent was waived by the research ethics boards of each participating centre for the original cohort. Approval for this secondary analysis of deidentified data was granted by the research ethics board of the centre holding the final dataset.

## RESULTS

A total of 1485 patients met the inclusion criteria, of whom 42 had incomplete echocardiographic data, 19 had structural heart defects, 4 had recurrent KD, 29 did not receive IVIG, and 1 was incorrectly diagnosed. There were 1382 patients included in the analysis. Of those, 812 (58.8%) were male and 570 (41.2%) females. Demographic parameters and treatment received were similar across groups (Table 1). The median age at diagnosis was 2.9 years old for males and 2.8 for females. Infants less than 1 year old at diagnosis represented 14.3% of the males and 13.6% of females. The percentage of incomplete KD was similar between sexes (31.8% of males and 29.2% of females).

**Table 1.** Baseline characteristics, with disease biological profile at onset and treatment received

	Males (n = 812)	Females (n = 570)	All (n = 1382)
Age at diagnosis year (median [IQR])	2.9 [1.5–5.0]	2.8 [1.6–4.8]	2.9 [1.5–4.9]
Age less than one year old at diagnosis (n, %)	116 (14.3)	77 (13.5)	193 (14.0)
Baseline laboratory results (median [IQR])*	112 [104–121]	113 [104–119]	113 [104–120]
Hemoglobin g/L	13.4 [10.0–17.0]	13.5 [10.1–17.4]	13.4 [10.05–17.14]
White blood cells × 109/L Platelets × 109/L	351 [264–459]	364.5 [275.5–477.0]	356 [270–466]
CRP mg/L	63 [29.0–129.0]	63 [31.9–188.2]	63 [30–126.2]
ESR mm/h	55 [40–79]	57 [43–85]	56 [41.0–82.0]
Albumin g/L	35 [31–39]	36 [32–40]	35 [31–39]
AST U/L	38 [29–55]	36 [28–54]	37 [28–54]
ALT U/L	31 [20–66]	28 [17.5–68]	29 [18–68]
Sodium mmol/L	137 [134–138]	137 [135–139]	137 [135–139]
Days of fever before treatment with IVIG (median [IQR])	6.0 [5.0–8.0]	6.0 [5.0–8.0]	6.0 [5.0–8.0]
Delayed treatment (>10 days) (n, %)**	74 (9.1)	59 (10.3)	133 (9.6)
Total fever duration (median [IQR])	8.0 [6.0–10.0]	7.0 [6.0–10.0]	7.0 [6.0–10.0]
Type of KD (clinical criteria met) (n, %)			
Complete	542 (68.2)	396 (70.8)	938 (67.9)
Incomplete	251 (31.8)	163 (29.2)	414 (30.0)
Unknown	19 (2.3)	11 (1.9)	30 (2.2)
Received more than one IVIG treatment (n, %)	212 (26.1)	137 (24.0)	349 (25.3)
Received other treatments (n, %)			
Oral corticosteroids	34 (4.2)	22 (3.9)	56 (4.1)
IV corticosteroids	62 (7.6)	34 (6.0)	96 (6.7)
Infliximab	4 (0.6)	1 (0.2)	5 (0.4)

*IVIG Intravenous Immunoglobulins*; *KD Kawasaki disease*.

*There were missing data for every variable: hemoglobin (376), white blood cells (315), platelets (320), albumin (560), sedimentation rate (387), C-reactive protein (421), ALT (421), AST (477), and sodium (481);

**data were missing for four participants.

### Risk of CAA according to the biological sex

The cumulative incidence of CAA was higher in males compared to females (Table 2). CAA was observed in 142 males (17.5%) and 55 females (9.7%). The adjusted risk difference was 6.0 percentage points (95% confidence interval [CI] 1.9 to 10.3). When we excluded small, isolated dilation of the LMCA from the analyses, we observed similar results (adjusted risk difference 6.3% CI 2.3% to 10.3%). A similar association between sex and medium or larger aneurysms (risk difference 4.6% CI 2.1 to 7.0) was observed. Giant aneurysms were also more likely to occur in males compared to females (risk difference 3.3% CI 1.7 to 5.8).

**Table 2.** Cumulative incidence of coronary aneurysm (CAA) as number of patients developing CAA (%) per category o, with adjacent risk differences

	Males (n = 812)	Females (n = 570)	Adjusted risk difference* Percentage point (95% CI)
Any CAA (Z ≥ 2.5)	142 (17.5%)	55 (9.7%)	6.0 (2.5–9.4)
CAA (Z ≥ 2.5)**	194 (23.9%)	93 (16.3%)	6.3 (2.3–10.3)
Medium or larger CAA (Z ≥ 5)	70 (8.6%)	19 (3.3%)	4.6 (2.1–7.0)
Giant CAA (Z ≥ 10)	37 (4.5%)	6 (1.1%)	3.3 (1.7–5.0)

*CAA coronary artery aneurysms*; *CI confidence interval*.

*Adjusted risk difference between males versus females;

**excluding small, isolated dilatation of the left main CA.

### Biological markers

**Figure 1.**
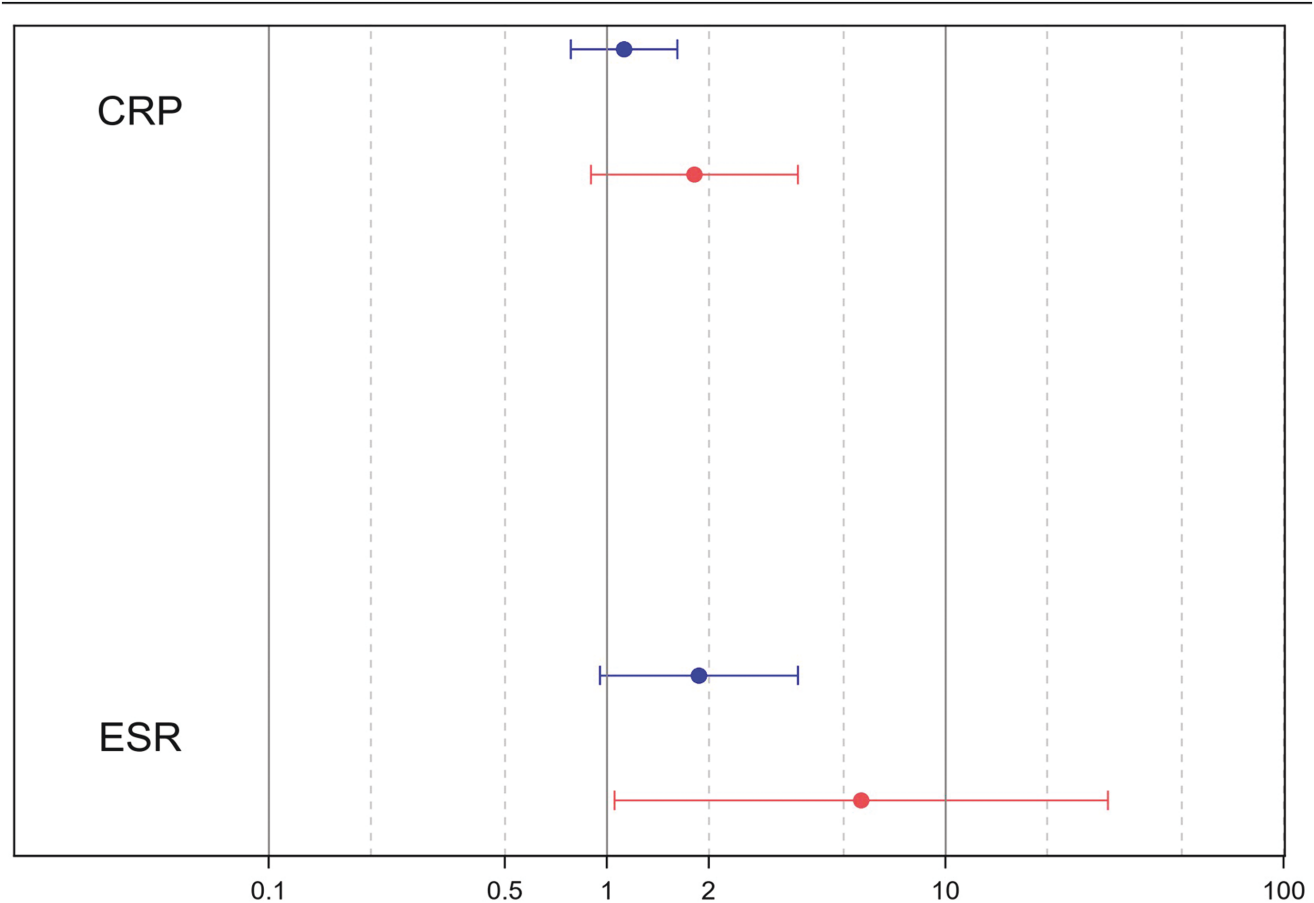
Odds ratio and 95%CI of the risk of coronary artery aneurysm (CAA) for each log increase of inflammatory marker according to biological sex. *CRP C-reactive protein; ESR erythrocyte sedimentation rate*

Higher CRP values were associated with an increased cumulative incidence of CAA, though not statistically significant, with each log increment in CRP corresponding to a 26% higher risk (CI 0.92 to 1.74). In contrast, each log increase in ESR was linked to a 2.30-fold higher risk of CAA (CI 1.20 to 4.28). Although graphical representation (Figure 1) suggested that the risk of CAA increased more significantly for females with an increase of both markers, the interaction terms with biological sex were not significant for either CRP (P = 0.24) or ESR (P = 0.23).

**Figure 2.**
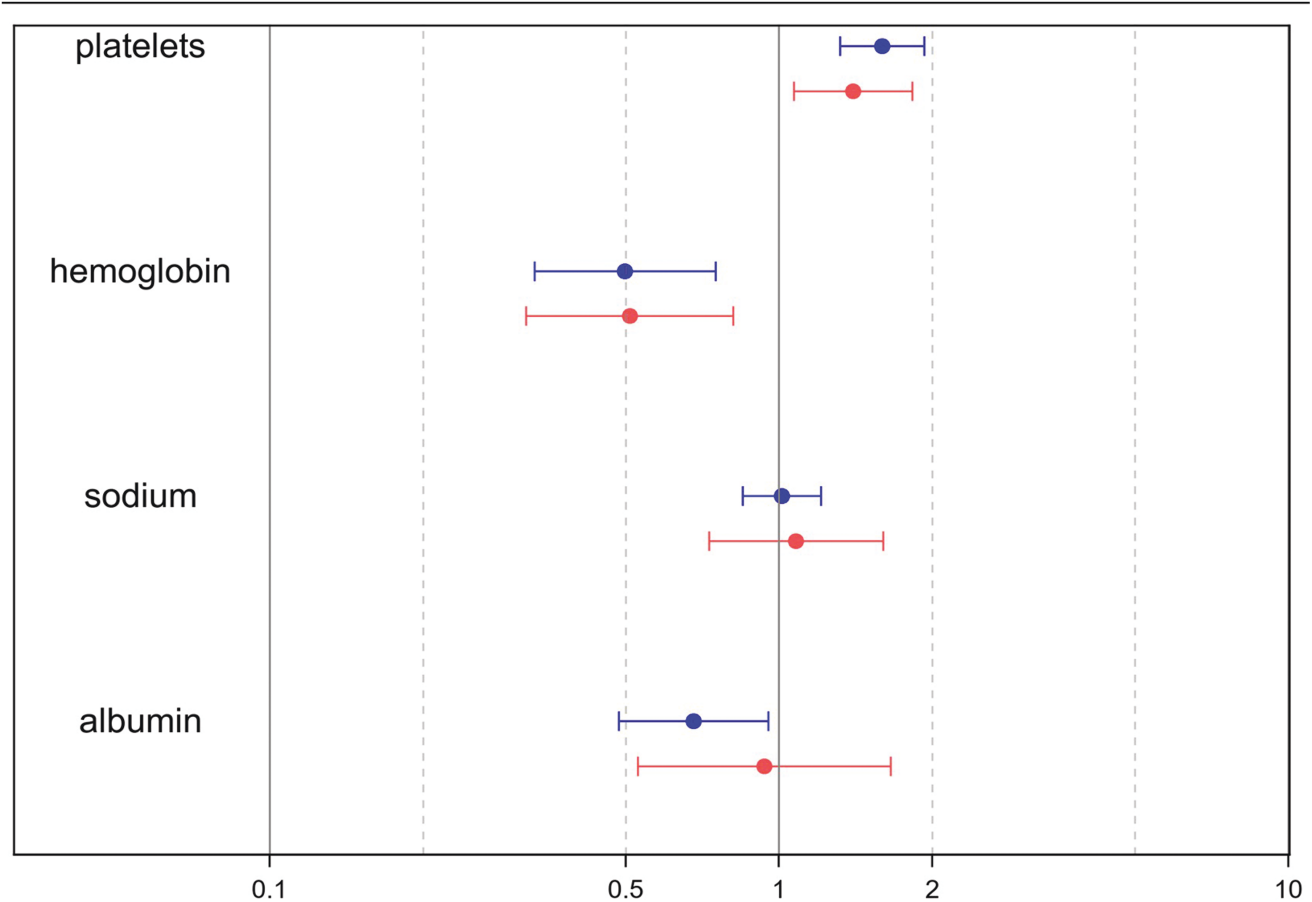
Odds ratio and 95% CI of the risk of developing a coronary artery aneurysm of any size according to biological sex. Data is shown for a variation of 2 standard deviations of the marker

Figure 2 shows the odds ratio of developing a CAA of any size based on variations in biological markers for both sexes. An increase of 2 standard deviations (SDs) in platelet count was linked to a higher risk of CAA (odds ratio [OR] 1.5, 95% CI 1.3 to 1.8), with similar effects in males (OR 1.6, 95% CI 1.3 to 1.9) and females (OR 1.4, 95% CI 1.1 to 1.8). The interaction term was not statistically significant (P = 0.43). Lower hemoglobin levels were associated with a higher risk of CAA (OR 2.0, 95% CI 1.5 to 2.7). A reduction of 2 SDs in hemoglobin level was associated with an equivalent increased risk for CAA in males (OR 2.0, 95% CI 1.3 to 3.0) and females (OR 2.0, 95% CI 1.2 to 3.1). The term of interaction was not statistically significant (P = 0.94). Lower albumin was associated with CAA development (OR 1.4, 95% CI 1.0 to 1.8) in general, but was associated with a definitive increased risk of CAA in males (OR 1.5 95% CI 1.1 to 2.1) while there was no statistically significant difference in females (OR 1.1 95%, CI 0.6 to 1.88). Nevertheless, the interaction term for sex was not statistically significant for this marker (P = 0.34). Finally, there was no association between lower sodium level and CAA risk (OR 1.0, 95% CI 0.8 to 1.2), and the interaction term for sex was not statistically significant (P = 0.77).

## DISCUSSION

KD is an acute vasculitis that primarily affects medium-sized blood vessels and is a leading cause of acquired heart disease in children. It is theorized that a potential antigen may activate both the innate and adaptive immune systems, leading to a cascade of inflammatory cells and circulating cytokines. Biological sex is a risk factor, with male patients exhibiting higher susceptibility to severe and complicated diseases (15). Our study confirms that aneurysms are more frequently observed in males than in females, with various severities of dilation linked to the male sex.

Multiple studies consistently demonstrate that males are at a greater risk for aneurysms of different sizes, as highlighted in a systematic review that reported an odds ratio of 1.75 for male sex (16). Additionally, Miura et al. (17) found that males are at an increased risk for complications related to coronary aneurysms. In 2017, Dietz et al. (18) identified male sex as a significant risk factor specifically for giant CAA (OR of 16.23). Furthermore, Porritt et al. reported that the cytokine IL-1β is significantly elevated in males with KD compared to females. This increased IL-1β expression can influence treatment responses, making males more likely to resist IVIG therapy and respond better to treatments targeting this cytokine, such as Anakinra (19). These findings emphasize the necessity of considering biological sex as a key determinant in assessing the predisposition to aneurysm formation associated with KD.

Our data indicated that inflammatory markers were associated with an increased risk of CAA. CRP is a known independent risk factor for coronary abnormalities in KD, in accordance with a retrospective study by Shuai et al. (20). The term of interaction was not significant for a modifying effect of sex in relation to ESR or CRP-associated risk of CAA, although we could hypothesize that females may be more affected by a comparable increase in these markers than males (based on effect size). It is possible that the sample size was insufficient to establish a modifying effect. This association may be attributed to a biological disparity in the inflammatory response between sexes. Notably, females may exhibit lower levels of inflammation compared to males (19). Thus, a substantial elevation in CRP and ESR could potentially signify a more severe disease manifestation in females. Further studies are warranted to validate this distinction.

Our study also demonstrated a positive correlation between platelet count and the development of CAA. Thrombocytosis has long been hypothesized as a predictor for coronary artery complications. Levin et al. (21) described the presence of thrombocytosis and immune complexes in the blood circulation during the third to the fifth week of the disease, highlighting a potential link between elevated platelet levels and the risk of coronary complications. Additionally, our study showed the presence of anemia as a predictor for CAA, as has been shown in other studies (22). Low albumin levels have also been demonstrated in our study to be a predictive factor for CAA. Low albumin levels reveal a state of severe inflammation, risk for coronary abnormalities (23), and IVIG resistance (23). For all these markers, no obvious sex-modifying effect was observed in our cohort.

### Strengths and limitations

Our study had several strengths. This is the first study to investigate biological sex-modifying effects on variations in inflammatory markers. Furthermore, our data originated from a well-characterized, extensive Canadian cohort, with follow-up information of minimally 6 weeks extending to up to 1 year post-diagnosis.

Our study has limitations. Several data elements, especially related to biological markers, were missing, potentially compromising the study’s statistical power and introducing biases. Laboratory values were documented at the time of diagnosis, precluding the consideration of marker fluctuations over time. Incomplete ethnicity data precluded the inclusion of this variable in our analyses, thus hindering our ability to ascertain whether our findings are applicable across all populations. We are aware that the sex assigned to prepubertal children is based on anatomy and that biological sex is a more complex interplay of anatomical, genetic, and hormonal influences. Biological sex cannot be simply reduced to two exclusive categories and there might be some overlapping between our two study groups. Nevertheless, sex ambiguity at birth remains a rare condition (~1/4500 births), which is not expected to affect our results (24).

## CONCLUSION

We found that KD affects males more than females and that males experience greater disease severity. While the etiology of this sex difference is unclear, studies highlight the role of specific inflammatory markers in immune response activation. The hormonal influence appears minimal, as nearly all patients were prepubertal at diagnosis; however, the role of sex hormones in older patients warrants further exploration. Inflammatory markers such as CRP, ESR, platelet count, hemoglobin, and albumin are key predictors of coronary artery aneurysm development, but we did not identify a significant modifying effect of biological sex. Future KD research should differentiate immune responses and lab values by sex for improved patient risk stratification and long-term prognostic evaluation (18).
